# European Perspective of 2-Person Rule for Biosafety Level 4 Laboratories

**DOI:** 10.3201/eid1511.091134

**Published:** 2009-11

**Authors:** Giuseppe Ippolito, Carla Nisii, Antonino Di Caro, David Brown, Robin Gopal, Roger Hewson, Graham Lloyd, Stephan Gunther, Markus Eickmann, Ali Mirazimi, Tuija Koivula, Marie-Claude Georges Courbot, Hervé Raoul, Maria R. Capobianchi

**Affiliations:** National Institute for Infectious Diseases, Rome, Italy (G. Ippolito, C. Nisii, A. Di Caro, M.R. Capobianchi); Health Protection Agency, London, UK (D. Brown, R. Gopal); Health Protection Agency, Salisbury, UK (R. Hewson, G. Lloyd); Bernhard Nocht Institute for Tropical Medicine, Hamburg, Germany (S. Gunther); Institute of Virology, Marburg, Germany (M. Eickmann); Swedish Institute for Infectious Disease Control, Solna, Sweden (A. Mirazimi, T. Koivula); French National Institute for Health and Medical Research, Lyon, France (M.-C. Georges Courbot, H. Raoul)

**Keywords:** Laboratories, safety management, safety, security measures, bioterrorism, biological toxins, letter

**To the Editor:** Recently, the directors of Biosafety Level 4 (BSL-4) laboratories in the United States published their views of the requirement of having ≥2 persons present at all times while biological work is undertaken in a BSL-4 laboratory (*1*). They concluded that safety and security would be better assured in some situations by video monitoring systems rather than by the presence of a fellow scientist. As members of the European Network of Biosafety Level-4 laboratories (Euronet-P4) who have developed guidelines in this area (*2*–*4*), we discussed the article during a recent network meeting. Biosafety and biosecurity are the major concerns for all involved in BSL-4 activities, and we support the authors’ initiative and broadly agree with their position. The consensus among European BSL-4 experts is that, in the interest of safety, standard practice should be for all laboratories to perform a risk assessment before any activity is undertaken. This preliminary assessment is the best way to determine procedures to be used, including whether 2 persons should work together as part of laboratory procedure. A 2-person rule is inappropriate simply because the best approach is not to have inflexible rules that are not objectively assessed according to laboratory-specific circumstances.

Surveillance video monitoring and data storing have their place in protecting laboratory facilities from unauthorized access and theft of materials, but their effectiveness for ensuring proper handling of pathogens is quite limited. Finally, we agree with the authors that both biosafety and biosecurity must be founded on careful selection and monitoring of staff, without which even the most sophisticated of control systems would fail.
